# Functional Diversity of Thalamic Reticular Subnetworks

**DOI:** 10.3389/fnsys.2018.00041

**Published:** 2018-10-18

**Authors:** John W. Crabtree

**Affiliations:** School of Physiology, Pharmacology, and Neuroscience, Medical Sciences Building, University of Bristol, Bristol, United Kingdom

**Keywords:** thalamic reticular nucleus, subnetworks, thalamic projection neurons, sensory thalamus, motor thalamus, intrathalamic interactions, attention

## Abstract

The activity of the GABAergic neurons of the thalamic reticular nucleus (TRN) has long been known to play important roles in modulating the flow of information through the thalamus and in generating changes in thalamic activity during transitions from wakefulness to sleep. Recently, technological advances have considerably expanded our understanding of the functional organization of TRN. These have identified an impressive array of functionally distinct subnetworks in TRN that participate in sensory, motor, and/or cognitive processes through their different functional connections with thalamic projection neurons. Accordingly, “first order” projection neurons receive “driver” inputs from subcortical sources and are usually connected to a densely distributed TRN subnetwork composed of multiple elongated neural clusters that are topographically organized and incorporate spatially corresponding electrically connected neurons—first order projection neurons are also connected to TRN subnetworks exhibiting different state-dependent activity profiles. “Higher order” projection neurons receive driver inputs from cortical layer 5 and are mainly connected to a densely distributed TRN subnetwork composed of multiple broad neural clusters that are non-topographically organized and incorporate spatially corresponding electrically connected neurons. And projection neurons receiving “driver-like” inputs from the superior colliculus or basal ganglia are connected to TRN subnetworks composed of either elongated or broad neural clusters. Furthermore, TRN subnetworks that mediate interactions among neurons within groups of thalamic nuclei are connected to all three types of thalamic projection neurons. In addition, several TRN subnetworks mediate various bottom-up, top-down, and internuclear attentional processes: some bottom-up and top-down attentional mechanisms are specifically related to first order projection neurons whereas internuclear attentional mechanisms engage all three types of projection neurons. The TRN subnetworks formed by elongated and broad neural clusters may act as templates to guide the operations of the TRN subnetworks related to attentional processes. In this review article, the evidence revealing the functional TRN subnetworks will be evaluated and will be discussed in relation to the functions of the various sensory and motor thalamic nuclei with which these subnetworks are connected.

## Introduction

**Figure 1:**
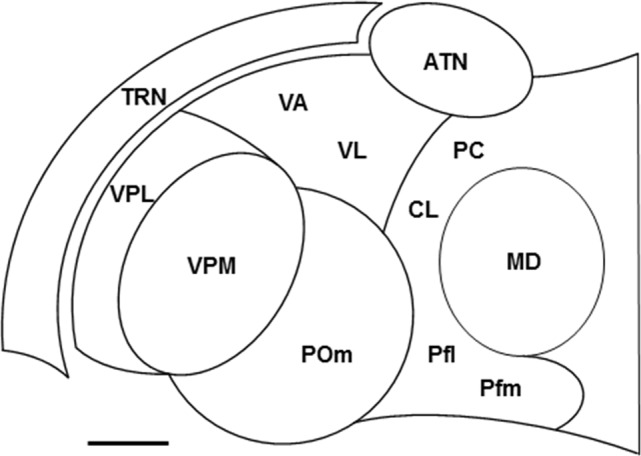
Locations of some nuclei of the thalamus. Schematic drawing of a horizontal section through the ventral part of the rat thalamus. Rostral is to the top and medial is to the right. Scale = 500 μm. Abbreviations: ATN, anterior thalamic nuclei; CL, centrolateral nucleus; MD, mediodorsal nucleus; PC, paracentral nucleus; Pfl, lateral part of parafascicular nucleus; Pfm, medial part of parafascicular nucleus; POm, posterior medial nucleus; TRN, thalamic reticular nucleus; VA, ventroanterior nucleus; VL, ventrolateral nucleus; VPL, ventroposterior lateral nucleus; VPM, ventroposterior medial nucleus. Other thalamic nuclei discussed in the text, the dorsal lateral geniculate nucleus, ventral medial geniculate nucleus, lateral posterior nucleus, and posterior lateral nucleus, are located dorsal to the section shown.

The thalamus is a prominent diencephalic structure that contains a large number of nuclei (Figure 1), each of which engages in its own specialized functions (Jones, 2007). This collection of nuclei is a major destination of pathways that carry exogenous, or stimulus-driven, information and those that carry endogenous, or internally generated, information. Whereas exogenous information travels through ascending sensory pathways such as the visual, auditory, and somatosensory systems, endogenous information arises from sensorimotor structures that, in a behavioral context, are links in cortical-subcortical networks that implement task-related, goal-directed, motor outputs. Along with the cerebral cortex, these structures include the cerebellum (CB; e.g., Middleton and Strick, 1998), basal ganglia (BG; e.g., Middleton and Strick, 2000; Hikosaka et al., 2006) and superior colliculus (SC; e.g., Krauzlis et al., 2013).

**Figure 2:**
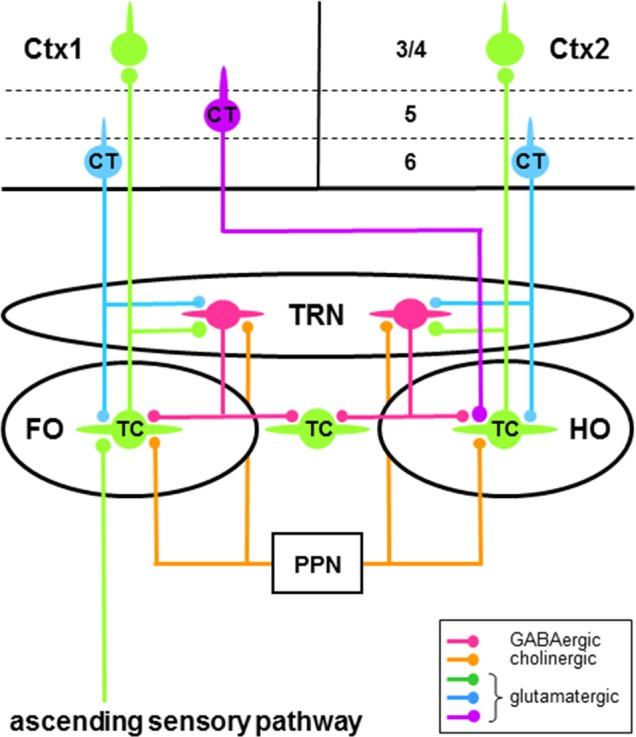
Schema of some neural circuitry of the thalamus, cortex and brainstem. Circuits showing first order (FO) and higher order (HO) connections of thalamocortical (TC) neurons (green cells) with neurons of the thalamic reticular nucleus (TRN; red cells) and cortical areas 1 (Ctx1) and 2 (Ctx2). Only the major thalamic-related input (layer 3/4) and output (layers 5 and 6) cortical laminae are shown. First order TC neurons receive driver afferents through ascending sensory pathways (green), whereas higher order TC neurons receive driver afferents through descending pathways (purple) from corticothalamic (CT) neurons in layer 5 (purple cell). Ascending and descending driver afferents have large axon terminals, whereas descending modulator afferents to TC and TRN neurons from CT neurons in layer 6 (blue cells) have small axon terminals. Note that TRN neurons can engage in closed-loop and/or open-loop circuits with TC neurons. Only brainstem modulator inputs (orange) from the pedunculopontine nucleus (PPN) are shown.

The main body of the thalamus is made up of nuclei composed of glutamatergic projection neurons and GABAergic interneurons (Sherman and Guillery, 2001; Jones, 2007). Most thalamic projection neurons are innervated by glutamatergic “driver” afferents (Guillery, 1995; Sherman and Guillery, 1998, 2001; Rovó et al., 2012), which make up a small proportion of these cells’ inputs, have large terminals containing round vesicles (RL type >2 μm in diameter), and provide the main information to be transmitted (e.g., receptive field properties). Depending on the origin of their driver afferents, thalamic projection neurons are defined as “first order” or “higher order” (Figure 2; Guillery, 1995; Sherman and Guillery, 1998, 2001; Sherman, 2016): first order projection neurons receive driver inputs from subcortical sources whereas higher order projection neurons receive driver inputs from corticothalamic (CT) neurons in layer 5 (L5) of cortex. The terminals of these inputs from L5 CT neurons and subcortical sources are immunoreactive to vesicular glutamate transporter type 1 (vGluT1) and type 2 (vGluT2), respectively (Rovó et al., 2012). A much larger proportion of the afferents to thalamic projection neurons are “modulator” inputs (Guillery, 1995; Sherman and Guillery, 1998, 2001; Sherman, 2016; see also Rovó et al., 2012), which alter the gain of the signal transmitted by these cells. These afferents mainly come from descending efferents of glutamatergic CT neurons in layer 6 (L6) of cortex (Figure 2) and have small terminals containing round vesicles (RS type <1 μm in diameter). Other sources of modulator inputs to thalamic projection neurons include the GABAergic neurons of the thalamic reticular nucleus (TRN; Figure 2) and the cholinergic neurons of the brainstem pedunculopontine nucleus (PPN; Figure 2) and basal forebrain (see McCormick, 1992 and Sherman and Guillery, 2001 for fuller accounts of the sources of modulator inputs to thalamic projection cells). Thalamic projection neurons send efferents to cortex—the thalamocortical (TC) cells (Figure 2)—or to the striatum—the thalamostriatal (TS) cells. The striatum (the caudate nucleus and putamen) is the major input structure of BG.

TRN is a sheet of GABAergic neurons (Houser et al., 1980; Oertel et al., 1983) that surround the main body of thalamic nuclei (Figure 1). Strategically located between the thalamic nuclei and cortex, these cells receive glutamatergic afferents from axon collaterals of TC neurons and L6 CT neurons (Figure 2), which provide driver (Gentet and Ulrich, 2003; Lam and Sherman, 2005, 2011, 2015) and modulator (Gentet and Ulrich, 2004) inputs, respectively. In addition to L6 CT afferents, TRN neurons also receive modulator inputs from many of the same sources that innervate thalamic projection neurons—for example, cholinergic inputs from PPN (Figure 2; see McCormick, 1992 and Sherman and Guillery, 2001 for fuller accounts of the sources of modulator inputs to TRN cells). TRN sends efferents to TC projection neurons, thus completing feedforward L6 CT→TRN→TC and feedback TC→TRN→TC inhibitory circuits (Figure 2; e.g., Salt, 1989; Kim and McCormick, 1998; Cruikshank et al., 2010). The feedback connections are mainly open-loop circuits (Pinault and Deschênes, 1998b; Crabtree, 1999; Pinault, 2004) that result in lateral inhibition of TC neurons (e.g., Salt, 1989; Binns et al., 2003; Lam and Sherman, 2005, 2015; Copeland et al., 2012). TRN afferents to TC neurons activate postsynaptic ionotropic GABA_A_ receptors and metabotropic GABA_B_ receptors that reduce neuronal responsiveness, respectively, through a non-hyperpolarizing shunting inhibition and a direct membrane hyperpolarization. As a major source of GABAergic inhibition in the thalamus, TRN has long been recognized as playing an important role in reducing the gain of the signal transmitted by thalamic projection neurons during tonic firing (Sherman and Koch, 1986; see Halassa and Acsády, 2016 for other sources of inhibition in the thalamus). Furthermore, TRN has long been associated with changes in thalamic activity during transitions from wakefulness to sleep (Steriade and Llinás, 1988; Lewis et al., 2015). Thus, rhythmic burst firing of TRN neurons and their interactions with TC neurons generate sleep-related oscillatory activity in the thalamus (McCormick and Bal, 1997; Fogerson and Huguenard, 2016; Halassa and Acsády, 2016). Note that the above circuits and processes would also apply to TS neurons.

Defined anatomically, TRN can be divided into several sectors that are distributed along the plane of the nucleus (Jones, 1975; Guillery et al., 1998; Crabtree, 1999; Guillery and Harting, 2003). These include a dorsocaudal visual sector (visTRN), a ventrocaudal auditory sector (audTRN), a ventrocentral somatosensory sector (ssTRN), and a rostral motor sector (mtrTRN)—including the “limbic” (Lozsádi, 1994, 1995; Halassa et al., 2014) and “(pre)frontal” (Zikopoulos and Barbas, 2006, 2012) sectors—and each of these sectors is connected to different thalamic nuclei and their associated cortical areas. Such anatomically defined sectors, with their delineation of borders, foster a compartmental view of the general organization of TRN. However, beyond this general view, mounting evidence shows that several functionally distinct subnetworks related to various sensory, motor, and cognitive processes operate across sectors within TRN.

Various experimental approaches have identified diverse, functionally distinct, subnetworks in TRN as defined by the spatial distributions, functional connections, and state-dependent activities of its constituent neurons. The purpose of this review is to focus on the identification of these subnetworks and their underlying circuitry and to show how they extend our current understanding of the functional roles of TRN in sensory, motor, and cognitive processes. Accordingly, a comprehensive and detailed description is provided to delineate the anatomical organization and function of the subnetworks, a topic not addressed by other recent reviews (Fogerson and Huguenard, 2016; Halassa and Acsády, 2016; Krol et al., 2018). To understand the functional significance of the TRN subnetworks, it will be important to take into account the functions of the thalamic nuclei with which the subnetworks are connected. Because these subnetworks often involve all or most of the neural population in a given TRN sector, many neurons contained therein most likely participate in two or more functionally distinct subnetworks. Such functional versatility would enable TRN neurons to differentially modulate thalamic projection neurons depending on ever-changing sensory, motor, and cognitive circumstances.

## Intra-TRN Subnetworks

### Chemical and Electrical Synapses

Previous electrophysiological studies indicated that TRN neurons are functionally connected through chemical (Sanchez-Vives et al., 1997; Shu and McCormick, 2002) and electrical (Landisman et al., 2002; Long et al., 2004) synapses. Such connectivity was subsequently mapped in *in vitro* slice preparations through ssTRN of young rats (10–15 days postnatal; Deleuze and Huguenard, 2006; Lam et al., 2006). Using laser-guided glutamate uncaging to activate neurons, inhibitory postsynaptic currents (IPSCs), mediated by GABA_A_ receptors (chemical synapses) and excitatory depolarizing spikelets, mediated by gap junctions (GJs; electrical synapses), were recorded in ssTRN neurons and were evoked from regions surrounding recorded cells—these regions are spatially restricted and generally correspond to the extent of the dendritic arbor of a recorded cell (Deleuze and Huguenard, 2006). Thus, in young rats, connected ssTRN neurons largely consist of two functionally distinct populations, one representing neurons that promote desynchronization of TRN activity through chemical synapses (Sohal and Huguenard, 2003) and the other representing neurons that promote synchronization of TRN activity through electrical synapses (Landisman et al., 2002; Long et al., 2004). However, in rodents older than 2 weeks of age, TRN neurons lose their inhibitory connections through chemical synapses (Landisman et al., 2002; Cruikshank et al., 2010; Hou et al., 2016) but retain their excitatory connections through electrical synapses (Landisman et al., 2002) and the ability to synchronize their activity (Long et al., 2004).

**Figure 3:**
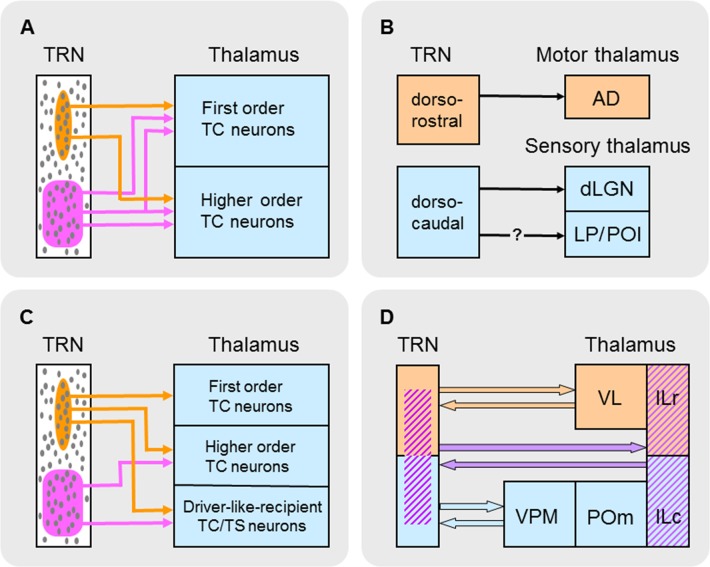
Schematic summary of different types of subnetworks in TRN. **(A)** TRN subnetworks identified according to their spatial distributions of electrically connected neurons through gap junctions (GJs) and connectivity with first order and/or higher order TC neurons. Elongated (circumscribed in orange) and broad (circumscribed in purple) GJ-related clusters of neurons (small gray ovals) are shown in TRN. **(B)** TRN subnetworks identified according to their state-dependent activity, location in TRN, and connectivity with first order thalamic nuclei. Neurons in dorsorostral TRN (orange) exhibit arousal-related activity whereas those in dorsocaudal TRN (blue) exhibit sleep-related activity. It is unclear whether this latter activity affects higher order sensory nuclei (LP and POl) in the thalamus. Abbreviations: AD, anterodorsal nucleus; dLGN, dorsal lateral geniculate nucleus; LP, lateral posterior nucleus; POl, posterior lateral nucleus. **(C)** TRN subnetworks identified according to their spatial distributions of neurons activated by glutamate corresponding to activation zones (AZs) and functional connectivity with first order and higher order TC neurons and driver-like-recipient thalamocortical/thalamostriatal (TC/TS) neurons. Elongated (circumscribed in orange) and broad (circumscribed in purple) AZ-related clusters of neurons (small gray ovals) are shown in TRN. **(D)** TRN subnetworks identified according to their mediation of interactions between groups of thalamic nuclei. Each group of interacting nuclei in the thalamus (shown on the right) is color coded. A possible distribution of neurons that mediate these interactions in TRN (shown on the left) is similarly color coded. Abbreviations: ILc, caudal intralaminar nuclei; ILr, rostral intralaminar nuclei; POm, posterior medial nucleus; TRN, thalamic reticular nucleus; VL, ventrolateral nucleus; VPM, ventroposterior medial nucleus.

In ssTRN of rodents, about one third to one half of the neural population form electrical synapses through GJs (Deleuze and Huguenard, 2006; Lam et al., 2006; Lee et al., 2014) and GJ-coupled clusters of these electrically connected neurons can be visualized through dye coupling following single-cell injections with neurobiotin (Lee et al., 2014)—TRN neurons number up to 24 cells in a cluster and average about nine cells per cluster. Defined in part by their spatial configurations, these GJ-coupled neural clusters mainly consist of two functionally distinct neural subnetworks (Figure 3A): about 63% of the clusters are elongated—the combined “elongated” and “discoid” types of clusters (Lee et al., 2014)—lie in the plane of TRN parallel to its borders, and occupy a fraction of its thickness, whereas about 14% of the clusters are broad—the “spherical” type of cluster (Lee et al., 2014)—occupy much of the thickness of TRN, and overlap the elongated clusters. The elongated clusters resemble the organizational components in TRN that represent local areas on a sensory surface (Crabtree, 1999). Thus, the elongated clusters would represent local areas on the somatosensory surface of the head or body and the broad clusters would represent more global, or multiple loci, on the somatosensory surface. The two main types of GJ-coupled neural clusters also differ in their projections to two prominent somatosensory regions in the thalamus, the ventroposterior medial (VPM) and ventroposterior lateral (VPL) nuclear complex and the posterior medial (POm) nucleus (Figure 1): whereas injected cells in elongated clusters project to either VPM/VPL, containing first order TC neurons, or POm, containing higher order TC neurons, injected cells in broad clusters project to either VPM/VPL or POm and can project to both VPM/VPL and POm through branching axons (Figure 3A). The different thalamic projections of the elongated and broad GJ-coupled clusters are consistent, respectively, with unbranched and branched projections from ssTRN seen in pathway tracing studies (rat: Pinault et al., 1995; cat: Crabtree, 1996). Although recordings and dye injections were restricted to ssTRN, the proportion of electrically connected neurons and their spatial configurations could be representative of the neural connectivity in other TRN sectors.

### Subnetworks Connecting TRN Sectors?

Involvement with a single sensory modality is usually thought to delineate neurons in the various sensory sectors of TRN (Guillery et al., 1998; Crabtree, 1999; Guillery and Harting, 2003). However, recent *in vivo* recordings in the adult rat show substantial cross-modal modulation of neural activity in visTRN and audTRN in response to visual and auditory stimuli (Kimura, 2014) and in audTRN and ssTRN in response to auditory and somatosensory stimuli (Kimura, 2017). Such cross-modal effects are subthreshold, predominantly suppress a response to a stimulus of one modality by a stimulus of another modality, and are present throughout an entire sensory sector of TRN. To account for these effects, it is tempting to attribute intra-TRN connectivity between sensory sectors—particularly inhibitory connections formed by chemical synapses—as the main underlying mechanism. However, after an early developmental period, all that remains of intra-TRN connections are those of electrical synapses; so, considering just the predominant cross-modal response suppression, how these excitatory connections could produce such suppressive effects is difficult to imagine. Alternatively, the widespread cross-modal modulation in a TRN sector suggests influences originating from extrathalamic sources, such as the widespread inputs to TRN from the cholinergic neurons of PPN (Figure 2; Hallanger et al., 1987; Steriade and Llinás, 1988; Winn, 2006)—these neurons receive afferents from multimodal cells in the intermediate/deep layers of SC (Steininger et al., 1992) and thus have short-latency multimodal responses to visual, auditory, and somatosensory stimuli (Winn, 2006; Gut and Winn, 2016). Cholinergic activation of TRN neurons results in fast and relatively weak excitatory postsynaptic currents (EPSCs), mediated by nicotinic (ionotropic) acetylcholine (ACh) receptors, followed by long-lasting and relatively strong IPSCs, mediated by muscarinic (metabotropic) ACh receptors (Sun et al., 2013). Accordingly, activation of cholinergic PPN→TRN circuits by stimuli of one modality could predominantly hyperpolarize TRN neurons through metabotropic ACh receptors, which would be well-suited to suppress responses to stimuli of another modality operating through glutamatergic TC→TRN circuits. Given appropriately timed onsets of stimuli of different modalities (Kimura, 2014, 2017), interactions between these modulator (cholinergic) and driver (glutamatergic) circuits could account for much of the observed cross-modal modulation of TRN neural activity. If so, PPN may play a role in rapidly informing TRN about the occurrence of potentially significant sensory stimuli during changing environmental circumstances (see Winn, 2006; Gut and Winn, 2016).

## TRN Subnetworks Related to Thalamocortical (TC) and Thalamostriatal (TS) Neurons

### First Order TC Neurons

Many first order TC neurons transmit ascending sensory information to cortex (Figure 2; Guillery, 1995; Sherman and Guillery, 1998; Sherman, 2016). The subcortical driver afferents to these cells are commonly branches of axons that also project to motor regions in the brainstem or spinal cord (Guillery and Sherman, 2002, 2011; Guillery, 2003, 2005; Sherman, 2016). According to this scheme, these driver afferents would carry two concurrent messages: information about sensory stimuli and copies of motor instructions, or efference copies, about potential or impending self-generated movements related to those stimuli. Efference copies sent to thalamus and then transmitted to cortex would continually update these higher brain areas about ongoing motor instructions sent to brainstem or spinal cord.

Two functionally distinct subnetworks of TRN neurons that connect with first order TC neurons have recently been identified (Figure 3B; Halassa et al., 2014; Chen et al., 2016). In the mouse, *in vivo* recordings from dorsocaudal TRN compared with those from dorsorostral TRN revealed different state-dependent patterns of neural activity. The activity of many neurons in the dorsocaudal region of TRN is positively correlated with sleep-related cortical rhythms (spindles and slow-waves). However, the activity of many neurons in the dorsorostral region of TRN increases upon arousal during wakefulness and is negatively correlated with sleep-related rhythms. Neurons in the dorsocaudal region of TRN send projections to a thalamic nucleus involved in sensory functions—in rodents, cells in this region are connected to first order TC neurons in the dorsal lateral geniculate nucleus (dLGN; Coleman and Mitrofanis, 1996), whose efferents convey visual information from the retina to visual cortex. Although recordings from this region (Halassa et al., 2014; Chen et al., 2016) did not distinguish between TRN neurons projecting to dLGN or higher order thalamic nuclei (the lateral posterior and posterior lateral nuclei; Coleman and Mitrofanis, 1996), it is most likely that many of the recorded cells projected to dLGN because such cells occupy the lateral two thirds of dorsocaudal TRN (Coleman and Mitrofanis, 1996) and would have been the first neurons encountered during the lateral approach of recording electrodes. In contrast, neurons in the dorsorostral region of TRN send projections to a thalamic nucleus involved in motor functions—in rodents, cells in this region are connected to first order TC neurons in the thalamic anterodorsal (AD) nucleus (Figure 1; Lozsádi, 1995; Petrof and Sherman, 2009) whose efferents carry navigational information during locomotor/exploratory behavior from the lateral mammillary nucleus of the hypothalamus to the postsubicular region of cortex (Blair et al., 1998). Driver afferents to TC neurons in AD are branches of axons that also descend to brainstem motor regions (Guillery and Sherman, 2002). In single-cell recordings in rats performing a food-retrieval task, AD neurons increase their firing rates when animals move their heads to face in a particular direction: individual AD neurons respond maximally to only one head direction and, collectively, head-direction preferences are distributed over the 360° range of possibilities (Blair and Sharp, 1995; Taube, 1995). These head-direction responses are abolished when animals are restrained and passively rotated (Taube, 1995), thus highlighting the primary contribution of efference copies (motor instructions) in determining the response properties of AD neurons. Together, the foregoing evidence suggests a sensory-motor dichotomy in TRN neural subnetworks based on their firing patterns during different behavioral states and their connectivity with first order TC neurons in either sensory or motor thalamic nuclei (Figure 3B).

Anatomical pathway tracing studies indicate that TRN neurons are topographically organized according to their efferent connections with first order TC neurons (Guillery et al., 1998; Crabtree, 1999; Guillery and Harting, 2003). Such neurons are found in VPM and VPL (Figure 1), whose efferents convey somatosensory information from the brainstem trigeminal complex (head representation) and dorsal column nuclei (body representation), respectively, to somatosensory cortex, and in the thalamic ventrolateral (VL) nucleus (Figure 1), whose efferents carry motor information from the output nuclei of CB (e.g., the dentate nucleus) to motor, premotor, and prefrontal cortical areas (monkey: Middleton and Strick, 1998, 2001; Haber and McFarland, 2001; rat: Kuramoto et al., 2009). Although VL efferents to cortex are widespread, these primarily target motor and premotor cortical areas. Furthermore, VL is a thalamic link in largely segregated Cortex→CB→VL→Cortex loops involved in motor control (monkey: Middleton and Strick, 1998, 2000)—for example, in monkeys performing visual tracking tasks, VL efferents carry information about sequential movements from the dentate nucleus to motor cortex. Using the glutamate-uncaging technique in *in vitro* slice preparations through the rodent thalamus, GABA_A_ receptor-mediated IPSCs were recorded in VPM and VPL neurons (rat: Lam and Sherman, 2005; mouse: Lam and Sherman, 2007) and VL neurons (mouse: Lam and Sherman, 2015) and were evoked from activation zones (AZs), or “footprints,” in ssTRN and mtrTRN, respectively. As noted earlier, using this technique activates neurons (Deleuze and Huguenard, 2006; Lam et al., 2006); therefore, the AZs and their spatial configurations correspond to underlying clusters of neurons activated by glutamate. Such clusters will hereafter be referred to as AZ-related clusters. Although data from VL and the functionally distinct thalamic ventroanterior (VA) nucleus were grouped together (Lam and Sherman, 2015), it appears that about two thirds of the cells were from VL—those recorded dorsocaudally—and the remaining cells were from VA—those recorded ventrorostrally—as defined using immunostaining techniques (Figure 1; Kuramoto et al., 2009, 2011). Single VPM, VPL, and VL neurons usually receive functional inputs from elongated AZ-related neural clusters in TRN that lie in the plane of the nucleus parallel to its borders—such clusters are distributed throughout the VPM-, VPL-, and VL-related territories. The AZ-related clusters in ssTRN shift mediolaterally or rostrocaudally in the outer (lateral) tiers of the thickness of TRN relative to similar shifts in the location of recorded VPM and VPL neurons, whereas the AZ-related clusters in mtrTRN shift mediolaterally or dorsoventrally in both the inner (medial) and outer tiers of the thickness of TRN relative to similar shifts in the location of recorded VL neurons. Thus, the corresponding shifts in clusters and recording locations indicate functional topographic organizations in TRN efferent connections with first order TC neurons. These functional maps are consistent with anatomically defined maps in ssTRN (rabbit: Crabtree, 1992a; cat: Crabtree, 1992b, 1996; rat: Pinault et al., 1995) and mtrTRN (rat: Cicirata et al., 1990).

### Higher Order TC Neurons

Higher order TC neurons transmit information from L5 of one cortical area to another cortical area through corticothalamocortical, or transthalamic, circuits (Figure 2; Guillery, 1995; Sherman and Guillery, 1998; Sherman, 2016). The cortical driver afferents to these cells are commonly branches of axons that also project to motor regions in brainstem or spinal cord (Deschênes et al., 1994; Guillery and Sherman, 2002, 2011; Guillery, 2003, 2005; Sherman, 2016). Accordingly, in addition to their sensory-related messages, these driver inputs would carry efference copies that would update the thalamus and the second cortical area about ongoing motor instructions that are generated by the first cortical area. Furthermore, through their interactions with cortex during goal-directed behavior, thalamic projection neurons involved in higher order circuits also play important roles in cognitive processes (Acsády, 2017; Halassa and Kastner, 2017; Nakajima and Halassa, 2017; Halassa, 2018; Rikhye et al., 2018), enabling (Purushothaman et al., 2012; Zhou et al., 2016) and sustaining (Bolkan et al., 2017; Guo et al., 2017; Schmitt et al., 2017) activity among neural networks within a cortical area and promoting neural synchrony across cortical areas (Saalmann et al., 2012; Zhou et al., 2016).

Anatomical pathway tracing studies indicate that TRN neurons are not topographically organized according to their efferent connections with higher order TC neurons (Guillery et al., 1998; Crabtree, 1999). Such neurons are found in POm (Figure 1), which conveys information through transthalamic circuits from somatosensory cortical area 1 to somatosensory cortical area 2 (mouse: Theyel et al., 2010); some POm neurons also receive convergent driver inputs from L5 of somatosensory cortex and the brainstem trigeminal complex (rat: Groh et al., 2014). Using the glutamate-uncaging technique in *in vitro* slice preparations through the mouse thalamus, GABA_A_ receptor-mediated IPSCs were recorded in POm neurons and were evoked from AZ-related neural clusters in ssTRN (Lam and Sherman, 2007). Single POm neurons receive functional inputs from AZ-related clusters with two different spatial configurations: about one third of the cells receive inputs from elongated clusters in the inner tier of the thickness of TRN—these clusters lie parallel to the inner border of the nucleus and are not topographically organized—whereas about two thirds of the cells receive inputs from broad clusters in TRN—these clusters span all or most of the thickness of the nucleus, are distributed throughout the territory connected to POm, and overlap the VPM- and VPL-related elongated clusters. The functional organization of this partly restricted but mainly broad TRN connectivity with higher order TC neurons is consistent with patterns seen in ssTRN in pathway tracing studies (rat: Pinault et al., 1995; Crabtree et al., 1998; cat: Crabtree, 1996).

### Thalamic TC/TS Neurons Receiving Driver-Like Inputs

**Figure 4:**
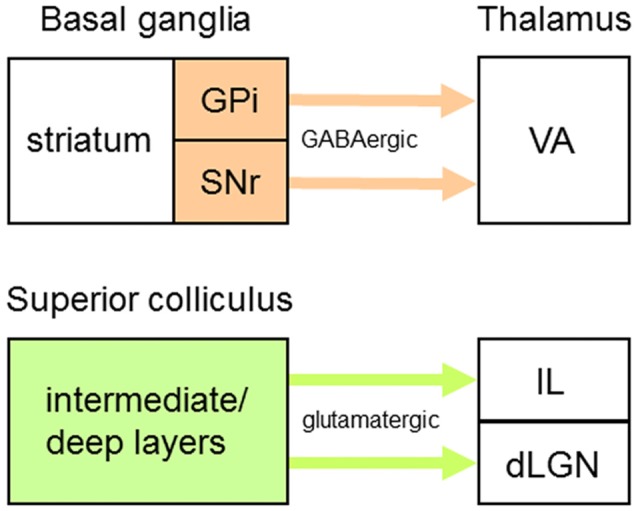
Origins of driver-like afferents to some thalamic nuclei. Driver-like inputs originating from output nuclei of the basal ganglia (orange) are GABAergic whereas those originating from output layers of the superior colliculus (green) are glutamatergic. Abbreviations: dLGN, dorsal lateral geniculate nucleus; GPi, internal globus pallidus; IL, intralaminar nuclei; SNr, substantia nigra pars reticulata; VA, ventroanterior nucleus.

Besides those that operate as first order or higher order TC neurons, some thalamic projection neurons transmit information provided by “driver-like” afferents—for example, GABAergic inputs from the output nuclei of BG, the substantia nigra pars reticulata (SNr) and internal globus pallidus (GPi) (Figure 4; Bosch-Bouju et al., 2013), or glutamatergic inputs from SC (Figure 4; Bickford, 2016) that have medium-sized RL-type terminals. In SC-recipient thalamic nuclei (e.g., dLGN), many driver-like inputs can converge on single projection neurons to determine their response properties (Smith et al., 2007; Bickford, 2016). In VA (Figure 1), a primary target of BG efferents, GABAergic afferents from SNr/GPi are widely distributed and have large terminals (>2 μm in diameter) with multiple synaptic release sites on somata and proximal dendrites of VA neurons (cat: Kultas-Ilinsky et al., 1985; monkey: Kultas-Ilinsky and Ilinsky, 1990; Ilinsky et al., 1997; Rovó et al., 2012). But are VA neurons also innervated by conventional driver inputs? When the thalamus of the monkey is immunoreacted for vesicular glutamate transporters, vGluT1- and vGluT2-positive RL-type terminals are not detected in VA (Rovó et al., 2012). However, there is evidence in the monkey that VA receives afferents from L5 of premotor (McFarland and Haber, 2002) and prefrontal (Zikopoulos and Barbas, 2007) cortical areas—inputs from prefrontal cortex correspond to anterogradely labeled terminals (mean diameter ± variance = 2.1 ± 0.44 μm) that form asymmetric synapses with (proximal?) dendrites of VA neurons. Thus, together with powerful driver-like (inhibitory) afferents from SNr/GPi, inputs from L5 CT neurons indicate the presence in VA of driver (excitatory) afferents from cortex, although they do not express a detectable level of vGluT1 or may use a different vesicular glutamate transporter. So, how can driver-like inhibitory inputs influence the action potential output of VA neurons? One long-standing possibility is that the firing rates of VA neurons could be shaped by transient and select inhibition of SNr/GPi outputs, thus producing transient and select disinhibition (or release from inhibition) of populations of VA neurons and a concomitant increase in their firing rates (Chevalier and Deniau, 1990; Mink, 1996).

Although the anatomical organization of TRN connections with neurons receiving driver-like inputs is unknown, the functional organization of projections from TRN to two thalamic nuclei containing such neurons, VA and the centrolateral (CL) nucleus (Figure 1), has been studied (Lam and Sherman, 2015). VA is predominantly a BG-recipient nucleus whose efferents carry motor information from the output nuclei of BG to striatum (rat: Kuramoto et al., 2009) and several cortical areas (monkey: Middleton and Strick, 2000, 2001; Haber and McFarland, 2001; rat: Kuramoto et al., 2009); although VA projections to cortex are widespread, these primarily target premotor and prefrontal cortical areas. Furthermore, VA is a thalamic link in Cortex→BG→VA→Cortex loops (monkey: Alexander et al., 1986; Alexander and Crutcher, 1990; Haber and McFarland, 2001) involved in selecting motor programs (Mink, 1996; Redgrave et al., 1999) and these loops contain positive (excitatory) feedback circuits—for example, in mice performing a tactile discrimination task, neurons in VA and premotor cortex engage in reverbatory interactions to sustain activity during preparation and selection of specific actions (Guo et al., 2017). CL is an SC-recipient nucleus (Chevalier and Deniau, 1984; Grunwerg and Krauthamer, 1992; Krout et al., 2001) whose efferents carry motor information from SC to striatum and motor and premotor cortical areas (see “TRN Subnetworks Related to Two or More Thalamic Nuclei” section for further elaboration of CL function). Using the glutamate-uncaging technique in *in vitro* slice preparations through the mouse thalamus, GABA_A_ receptor-mediated IPSCs were recorded in VA and CL neurons and were evoked from AZ-related neural clusters in mtrTRN (Lam and Sherman, 2015). About one half of the VA neurons and most CL neurons receive functional inputs from elongated AZ-related clusters in TRN that lie in the plane of the nucleus parallel to its borders—such clusters are distributed throughout the VA- and CL-related territories. The elongated clusters shift mediolaterally or dorsoventrally in mainly the outer tier of the thickness of TRN relative to similar shifts in the location of recorded VA neurons, whereas such clusters shift dorsoventrally in mainly the inner tier of the thickness of TRN relative to similar shifts in the location of recorded CL neurons. Thus, the corresponding shifts in clusters and recording locations indicate functional topographic organizations in TRN efferent connections with TC/TS neurons receiving driver-like inputs; these functional maps are consistent with the organization of an anatomically defined map in mtrTRN related to VL (rat: Cicirata et al., 1990). However, about one half of the VA neurons receive functional inputs from broad AZ-related clusters in TRN that span all or most of the thickness of the nucleus—these clusters are distributed throughout the territory connected to VA and overlap the VA-related elongated clusters.

**Figure 5:**
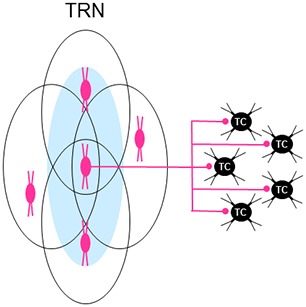
Individual neurons in the TRN are part of multiple overlapping neural clusters. Related to a particular type of thalamic projection neuron, individual reticular neurons in a neural cluster (e.g., central red cell of three cells shown underlying an activation zone (AZ; blue oval) shown on the left) are also part of other AZ-related neural clusters (outlined ovals) formed by neighboring reticular neurons, creating a dense subnetwork of multiple overlapping neural clusters in TRN. This anatomical arrangement would apply to both elongated and broad types of AZ-related clusters of reticular neurons (see text for details). Because each reticular neuron usually provides inhibitory inputs to several neighboring thalamocortical (TC) neurons (only five are shown on the right), the divergent outputs of reticular neurons suggest that single TC neurons can receive convergent inhibitory inputs from neurons in more than one neural cluster in TRN.

In summary, two functionally distinct subnetworks of TRN neurons have been identified according to their firing patterns during different behavioral states—sleep and arousal (Figure 3B); these sleep- and arousal-related subnetworks are connected, respectively, with first order TC neurons in sensory (dLGN) and motor (AD) thalamic nuclei. Furthermore, two other functionally distinct TRN subnetworks have been identified that are made up of AZ-related clusters of neurons with different spatial configurations—elongated and broad (Figure 3C). Individual clusters provide convergent inhibitory inputs onto single TC and TC/TS neurons in sensory and motor nuclei. The elongated and broad AZ-related clusters within a TRN sector are functionally connected to particular types of projection neurons in various thalamic nuclei: elongated clusters provide local topographical modulation of first order TC neurons in somatosensory (VPM and VPL) and motor (VL) nuclei, BG-recipient TC/TS neurons in a motor nucleus (VA), and SC-recipient TC/TS neurons in a motor nucleus (CL), whereas broad clusters provide more global non-topographical modulation of higher order TC neurons in a somatosensory nucleus (POm) and BG-recipient TC/TS neurons in a motor nucleus (VA). Because individual TRN neurons in either elongated or broad AZ-related neural clusters are also part of similarly configured clusters related to neighboring cells (Figure 5), a picture emerges of two functionally distinct and dense subnetworks in TRN each of which is composed of multiple overlapping neural clusters that are widely distributed within a TRN sector—although segregated, these two subnetworks can overlap. Despite an experimental approach that emphasized the functional connections of individual AZ-related clusters in TRN with single thalamic projection neurons (Lam and Sherman, 2005, 2007, 2015), these clusters should not be thought of as engaging these neurons in a 1:1 relationship—such a scheme cannot account for how a small population of neurons in a TRN sector supplies inhibition to a much larger population of neurons in a thalamic nucleus. Instead, individual TRN neurons usually provide divergent inhibitory inputs onto several neighboring thalamic projection neurons (Figure 5; Salt, 1989; Pinault et al., 1995; Cox et al., 1996; Pinault and Deschênes, 1998a,b; Binns et al., 2003; Lam and Sherman, 2005, 2015; Copeland et al., 2012). For the two projection neuron-connected subnetworks in TRN—formed by elongated or broad AZ-related neural clusters—the spatial configurations of these clusters are strikingly similar to GJ-coupled clusters of electrically connected TRN neurons (Figure 3A; Lee et al., 2014). Because every neuron in a TRN sector participates in one or the other projection neuron-connected subnetwork, each subnetwork most likely incorporates all of the spatially corresponding electrically connected neurons. Synchronous activity through electrical connections in the projection neuron-connected subnetworks would enhance the impact of their convergent inhibitory inputs onto TC and TC/TS neurons.

## TRN Subnetworks Related to Two or More Thalamic Nuclei

Because of the absence of direct glutamatergic connections between projection neurons in different thalamic nuclei, these cells are thought to have their own private lines of communication with cortex and/or striatum. However, anatomical pathway tracing studies suggested that projection neurons in different thalamic nuclei could functionally interact through disynaptic pathways mediated by TRN. Thus, thalamoreticular terminals and/or TRN neurons related to two or more thalamic nuclei are often situated in close proximity within overlapping territories (Cicirata et al., 1990; Pinault et al., 1995; Crabtree, 1996, 1998; Kolmac and Mitrofanis, 1997; Crabtree et al., 1998; Pinault and Deschênes, 1998a), TRN neurons can project to more than one thalamic nucleus (Pinault et al., 1995; Crabtree, 1996, 1998; Kolmac and Mitrofanis, 1997), and axonal fasciculi that stream across the thalamus have parallel trajectories that relate to particular subsets of thalamic nuclei and TRN regions (Crabtree and Isaac, 2002). Each of these anatomical factors can contribute to the creation of interfaces for TRN-mediated interactions between projection neurons in different thalamic nuclei. This possibility was tested in an *in vitro* slice preparation taken in the horizontal plane through the rat thalamus (Figure 1; Crabtree et al., 1998; Crabtree and Isaac, 2002). Robust GABA_A_ receptor-mediated IPSCs were recorded from single neurons in one thalamic nucleus evoked by activating neurons with locally applied glutamate in another thalamic nucleus. Thus, through disynaptic open-loop TC→TRN→TC circuits (Figure 2), activation of neurons in one thalamic nucleus can inhibit and temporarily interrupt tonic firing of neurons in another thalamic nucleus; note that the above circuits would also apply to TS neurons. In our slice preparation, these circuits mediate reciprocal interactions within particular groups of thalamic nuclei—the caudal and rostral intralaminar (ILc and ILr, respectively) nuclei, VPM, POm, and ILc, and VL and ILr—but not within other groups of nuclei (e.g., VPM/POm and VL). Furthermore, when neurons in two or more thalamic nuclei are activated, convergence of inhibitory inputs on single thalamic neurons can also occur across groups—for example, TRN-mediated inhibition of ILr neurons following activation of VL and ILc neurons or of ILc neurons following activation of VPM, POm and ILr neurons. Reciprocal interactions between pairs of nuclei show a general caudal to rostral topographic organization between activation and recording sites, reflecting the parallel organization of the axonal fasciculi that traverse the thalamus.

### Caudal and Rostral Intralaminar Nuclei

Located medially within the internal medullary lamina, ILc and ILr are groups of thalamic nuclei whose topographically organized reciprocal interactions are most likely mediated by neurons in a broad central to rostral region of TRN (Kolmac and Mitrofanis, 1997; Crabtree and Isaac, 2002). In the rodent, ILc mainly includes the lateral and medial parts of the parafascicular (Pfl and Pfm, respectively) nucleus (Figure 1)—the centromedian (CM) and Pf nuclei in monkeys are homologous, respectively, to Pfl and Pfm in rodents—and ILr mainly includes CL and the paracentral (PC) nucleus (Figure 1). ILc and ILr are both SC-recipient nuclear complexes (Figure 4; McHaffie et al., 2005) receiving afferents from multimodal cells in the intermediate/deep layers of SC (Grunwerg and Krauthamer, 1992; Krout et al., 2001); for example, these inputs to ILr have vGluT2-immunoreactive medium-sized RL-type terminals (Rovó et al., 2012). SC afferents to ILc and ILr are commonly branches of axons that also project to motor regions in other brainstem areas or spinal cord (Chevalier and Deniau, 1984); so, these inputs would carry efference copies that inform ILc and ILr about ongoing motor instructions that are generated by SC. ILc and ILr efferents carry information about sensory events and motor events, respectively, to striatum and motor and premotor cortical areas through branching axons (Deschênes et al., 1996a,b; Parent and Parent, 2005). Although ILc neurons respond to stimuli of a single sensory modality (visual, auditory, or somatosensory) or to combinations of stimuli of different modalities (Peschanski et al., 1981; Grunwerg and Krauthamer, 1992; Matsumoto et al., 2001), these cells are not engaged in detailed analyses of sensory stimuli. Instead, in single-cell recordings in monkeys undergoing classical conditioning or performing a visually guided task, CM/Pf neurons register the occurrence of unexpected sensory stimuli (Matsumoto et al., 2001; Minamimoto et al., 2005, 2014) or the occurrence of relevant (reward-associated) sensory stimuli (Matsumoto et al., 2001; Minamimoto and Kimura, 2002)—responses to sensory stimuli do not habituate when paired with reward—indicating roles for these cells in attentional orienting, behavioral switching, and sensorimotor learning (see “TRN Subnetworks Related to Cognition” section for further elaboration of ILc function). In single-cell recordings in monkeys performing oculomotor tasks, ILr neurons register the occurrence of task-relevant visual stimuli (Schlag-Rey and Schlag, 1984), visually evoked saccades (Schlag and Schlag-Rey, 1984), both of these events (Wyder et al., 2003; 44% of recorded cells), or those related exclusively to saccades (Wyder et al., 2003; 50% of recorded cells). Eye movement-related changes in firing rates of ILr neurons, during both impending and actual saccades, thus highlight the contribution of efference copies (motor instructions) in determining the response properties of these cells.

### Somatosensory and Caudal Intralaminar Nuclei

VPM, POm and ILc make up a caudal group of thalamic nuclei (Figure 1) whose topographically organized reciprocal interactions are most likely mediated by neurons in a broad central region of TRN (Kolmac and Mitrofanis, 1997; Crabtree et al., 1998; Crabtree and Isaac, 2002). Each of the caudal group of interacting nuclei, VPM, POm and ILc, contains projection neurons engaged in transmitting information arising from different origins, an ascending sensory pathway, L5 CT neurons, and SC, respectively. As noted earlier, VPM conveys somatosensory information to cortex, POm conveys information between cortical areas, and ILc conveys information about sensory events to striatum and cortex, The number of neurons in the TRN subnetwork underlying interactions between VPM, POm and ILc neurons can be partly gauged from* in vitro* recordings of EPSCs in the mouse ssTRN evoked by glutamate activation of VPM/VPL and POm neurons (Lam and Sherman, 2011): approximately a fifth of the recorded TRN neurons receive converging inputs from VPM/VPL and POm neurons, providing a possible anatomical substrate to account for their interactions. Including glutamate activation of ILc neurons in such recordings will most likely increase the estimated size of the TRN neural subnetwork mediating interactions between VPM, POm and ILc.

### Motor and Rostral Intralaminar Nuclei

VL and ILr form a rostral group of thalamic nuclei (Figure 1) whose topographically organized reciprocal interactions are most likely mediated by neurons in a rostral region of TRN (Cicirata et al., 1990; Kolmac and Mitrofanis, 1997; Crabtree and Isaac, 2002); as previously noted, efferents of VL and ILr carry motor information to striatum and/or cortex. Each of the rostral group of interacting nuclei, VL and ILr, contains projection neurons engaged in transmitting information that arises from different origins, CB and SC, respectively. However, the number of neurons in the TRN subnetwork underlying interactions between VA/VL and CL (part of ILr) was very small as gauged from experiments in a slice preparation taken in an oblique coronal plane through the mouse thalamus (Lam and Sherman, 2015). The results of this recent study are in sharp contrast to those of an earlier study (Crabtree and Isaac, 2002) in which interactions between VL and ILr were readily observed and topographically mapped, suggesting instead that the size of the TRN neural subnetwork underlying these interactions is substantial. Differences in experimental procedures between the earlier and latter studies may account for the different outcomes. For example, in the earlier study, slice preparations were taken in the horizontal plane to optimize the incorporation of intact axonal fasciculi that traverse the thalamus. Furthermore, activation and recording sites in ILr included PC but were restricted to VL in the VA/VL thalamic region. VA is probably part of another group of interacting thalamic nuclei that includes the mediodorsal (MD) nucleus but not CL. Like VA, MD receives afferents from BG and is reciprocally connected with prefrontal cortex (Vertes, 2006; Mitchell and Chakraborty, 2013; Collins et al., 2018)—the territories occupied by TRN neurons projecting to VA or MD largely overlap and some of these cells project to both VA and MD (Zikopoulos and Barbas, 2006).

In summary, thalamic nuclei can functionally interact through TRN-mediated disynaptic TC/TS→TRN→TC/TS circuits. Thus, activation of neurons in one nucleus results in GABA_A_ receptor-mediated inhibition and temporary interruption of tonic firing of neurons in another nucleus. Interactions between pairs of thalamic nuclei are reciprocal and provide ample opportunity for intramodality and intermodality modulation of transmission through the thalamus. The TRN subnetworks that mediate such interactions (Figure 3D) are not widespread but are topographically organized and link together neurons in homologous regions within specific groups of nuclei: ILc and ILr, VPM, POm and ILc, and VL and ILr. Along with their possible transmission of efference copies (motor instructions), groups of interacting thalamic nuclei are related to either sensory processing—VPM, POm and ILc—or motor processing—VL and ILr—whereas the ILc and ILr group bridges these sensory and motor operations. These thalamic nuclei convey different messages that represent different stages of sensory or motor processing—detection of events without analysis (ILc and ILr), detailed thalamic analysis (VPM and VL), or detailed cortical analysis (POm). The neural subnetworks in TRN underlying the interactions within thalamic groups may not be entirely segregated but could involve some of the same cells. Thus, TRN neurons related to ILc occupy much of the territory of those related to VPM/POm (Figure 3D; Kolmac and Mitrofanis, 1997; Crabtree et al., 1998) and TRN neurons related to ILr occupy much of the territory of those related to VL (Figure 3D; Cicirata et al., 1990; Kolmac and Mitrofanis, 1997). Therefore, it is not surprising that, besides forming their own group of interacting nuclei, ILc and ILr are each part of another group of interacting nuclei and thus are targets of TRN-mediated convergence of inhibition following activation of neurons in two or more thalamic nuclei.

## TRN Subnetworks Related to Cognition

Attention is a cognitive function that involves covert changes in neural representations of sensory stimuli in the absence of overt changes in behavior (e.g., head or eye movements). Because at any given moment the amount of sensory information available in the external environment far exceeds the processing capability of the brain, attentional mechanisms allow access to limited neural resources to select a small fraction of this information. Such attentional selection is controlled through exogenous “bottom-up” processes and endogenous “top-down” processes (e.g., Corbetta and Shulman, 2002; Fecteau and Munoz, 2006; Awh et al., 2012; Buschman and Kastner, 2015; Womelsdorf and Everling, 2015). Bottom-up attentional control is determined by the physical salience of a stimulus resulting in a stimulus-driven selection process. Top-down attentional control is determined by previous experiences and expectations: when associated with reward during behavioral tasks, top-down attentional selection is determined by the relevance of a stimulus to current goals resulting in a goal-driven selection process. However, attentional control biases due to previous selection and reward histories must also be taken into account when considering top-down attentional processes (Awh et al., 2012; Womelsdorf and Everling, 2015). This general scheme of stimulus-driven and goal-driven attentional selection can be readily applied to information processing in the thalamus.

Because of its unique combination of properties—positional, connectional, physiological, and neurochemical—TRN has long been thought to play a role in attention (Crick, 1984; Guillery et al., 1998; Pinault, 2004). This view is consistent with the strong contribution that the inhibitory neurons of TRN make in gating thalamocortical transmission (Sherman and Koch, 1986) and with the evidence that TRN connections with some thalamic nuclei—those containing first order TC neurons—and their associated cortical areas are topographically organized (Guillery et al., 1998; Crabtree, 1999; Guillery and Harting, 2003). Early behavioral tests of bottom-up (Weese et al., 1999) and top-down (McAlonan et al., 2000) stimulus selection in the rat found that TRN is indeed involved in attentional processes. To implement these processes, activation of TRN neurons by glutamatergic driver inputs is required. This requirement is met in the bottom-up condition by driver TC→TRN circuits (Figure 2) but descending (top-down) L6 CT afferents are usually considered to be modulators (Guillery, 1995; Sherman and Guillery, 1998, 2001; Guillery and Harting, 2003; Gentet and Ulrich, 2004) and thus would not be expected to activate TRN neurons. However, under conditions of synchronous activity, convergent glutamatergic inputs from L6 CT neurons are capable of producing action potentials in TRN neurons (Landisman and Connors, 2007; Cruikshank et al., 2010; Lam and Sherman, 2010; Paz et al., 2011) and thus can act like driver inputs.

### Bottom-Up Attentional Processes

As determined by the salience of a stimulus, bottom-up attentional processes play a prominent role in sensory detection and discrimination. To examine such processes in TRN, one line of enquiry has focused on synaptic interactions between VPM—containing first order TC neurons—and ssTRN in the rat (Salt and Eaton, 1995; Salt and Turner, 1998; Binns et al., 2003; Turner and Salt, 2003; Copeland et al., 2012); in rodents, both VPM (Van der Loos, 1976) and ssTRN (Shosaku et al., 1984) contain a particularly large vibrissal (whisker) representation. VPM neurons were recorded *in vitro* during combined electrical stimulation of TRN and bath-applied compounds or recorded *in vivo* during combined whisker stimulation and local application of compounds at recording sites. In feedback VPM→ssTRN→VPM circuits (Figure 2), the effects of various compounds on inhibition arising from ssTRN were assessed in VPM neurons before and during drug application by recording inhibitory postsynaptic potentials (IPSPs) and whisker-evoked tonic firing. Activation of presynaptic glutamate receptors on ssTRN axon terminals—involving Groups II and III metabotropic receptors and ionotropic kainate receptors—results in reduced release of GABA from these terminals and a subsequent attenuation of feedback inhibition of VPM neurons; thus, IPSP amplitudes are reduced and tonic firing of VPM neurons increases. Under physiological conditions, these presynaptic receptors are most likely activated by spillover of glutamate released at synapses on VPM neurons during their activation by ascending sensory afferents. Accordingly, glutamate receptors would be activated on terminals of ssTRN neurons engaged in either closed-loop (recurrent) or open-loop (lateral) inhibition of VPM neurons. However, attenuation of feedback inhibition of VPM neurons would be particularly pronounced if  VPM and ssTRN neurons have the same dominant receptive field center (e.g., primarily driven by the same principal whisker) and engage in closed-loop circuits. Thus, attenuation of inhibition in closed-loop VPM→ssTRN→VPM circuits (Figure 2) provides a local attentional mechanism that promotes sensory discrimination (Salt, 2002; Binns et al., 2003)—the firing rate of a stimulus-driven VPM neuron would be relatively enhanced compared to the firing rates of neighboring VPM neurons.

### Top-Down Attentional Processes

As determined by the reward-associated relevance of a stimulus to current behavioral goals, top-down attentional processes also play a prominent role in sensory detection and discrimination. To examine such processes in TRN, recent studies have focused on the activity of neurons in visTRN (McAlonan et al., 2008; Halassa et al., 2014; Wimmer et al., 2015; Chen et al., 2016). Single cells were recorded in dLGN—containing first order TC neurons—and visTRN in monkeys performing an oculomotor task in which saccades were made to visual stimuli or central fixation was maintained (McAlonan et al., 2008); correct responses were rewarded. During trials, the activity of neurons in visTRN is modulated in a time- and location-dependent manner: when two stimuli are presented, tonic firing is reduced for stimuli lying within the receptive fields of recorded cells (“ATTin”) compared to stimuli lying outside their receptive fields (“ATTout”). Conversely, under the same experimental conditions, dLGN neurons increase their firing rate. Thus, after presentation of stimuli, modulation of visTRN neurons (reduced firing) precedes that of dLGN neurons (increased firing) consistent with a role for visTRN in visual attention. As to the source of this modulation in the monkey, the projection to TRN from the dorsolateral prefrontal cortex (DLPFC; Zikopoulos and Barbas, 2006)—neurons here participate in cognitive functions (Vertes, 2006)—may provide the anatomical basis for the attentional modulation. However, the terminals of this projection in TRN are mainly concentrated in mtrTRN and do not reach the dorsocaudal visTRN. L6 CT neurons that project to visTRN specifically originate from visual cortical areas (Guillery et al., 1998; Crabtree, 1999; Guillery and Harting, 2003) and this specificity strongly suggests that visual cortex must be involved in attentional modulation of neurons in visTRN.

Furthermore, single cells were recorded in dLGN and visTRN of mice performing a visual detection task (Halassa et al., 2014; Chen et al., 2016) or a rule-specific (“attend to vision” or “attend to audition”) discrimination task (Wimmer et al., 2015) in which leftward or rightward movements were made to the locations of visual or auditory stimuli; correct responses were rewarded. During visual detection trials and attend to vision trials, tonic firing of neurons in visTRN is reduced during a stimulus “anticipation” period prior to stimulus presentation. This modulation—a reduction in the inhibitory output of visTRN neurons—is causally linked to latency and accuracy of responses in the tasks and produces an increase in the firing rate of dLGN neurons specifically through feedforward L6 CT→visTRN→dLGN circuits (Figure 2). Furthermore, an optogenetic manipulation (light spread of about 600 μm) was used to disrupt function in the prelimbic prefrontal cortex (PLPFC)—in the rodent, PLPFC is involved in cognitive functions and is homologous to DLPFC in the monkey (Vertes, 2006); such disruption during the stimulus anticipation period diminishes the attentional modulation of neurons in visTRN and the accuracy of stimulus detection in the attend to vision task. These results indicate that, during attentional processes, PLPFC exerts a strong influence on neurons in visTRN, possibly through a direct pathway. However, in rodents, PLPFC afferents to TRN are restricted to mtrTRN (Vertes, 2002); so, if the dorsocaudal visTRN is not a target of PLPFC inputs, through what connectional route does the mouse PLPFC influence dLGN-connected neurons in visTRN? In rodents, these neurons occupy the lateral two thirds of this sector (Coleman and Mitrofanis, 1996) and they receive L6 CT inputs that specifically arise from visual cortical areas, which include areas 1 (V1) and the medial part of 2 (V2m) that flanks V1 medially (Coleman and Mitrofanis, 1996; Wang and Burkhalter, 2007). So, L6 CT neurons in V1 and V2m must be involved in attentional modulation of dLGN-connected neurons in visTRN—together, V1 and V2m extend approximately 3.0 mm rostrocaudally and 2.0 mm mediolaterally and contain three representations of the visual field. However, using the same optogenetic manipulation as before, disrupting function in V1 during stimulus anticipation does not diminish the accuracy of stimulus detection in the attend to vision task; but this result is not surprising given that the optogenetic manipulation perturbed only a small fraction (about 5%) of the total V1 and V2m cortical area leaving neurons in the vast majority of this area functionally unperturbed and capable of mediating top-down attentional processes. Thus, involvement of V1 and V2m cannot be ruled out in a PLPFC-originating pathway through which attentional processes can influence neurons in visTRN. Because PLPFC does not project directly to V1 and V2m in rodents (Vertes, 2006), this pathway most likely involves disynaptic corticocortical connections from PLPFC to V1 and V2m (Nguyen et al., 2015) before reaching visTRN.

Combining genetic manipulations and behavioral testing, a recent study in the mouse has revealed a role for ErbB4 in top-down attentional mechanisms in the thalamus (Ahrens et al., 2015)—ErbB4 is a neuregulin-1 (NRG1) receptor expressed in somatostatin-positive (SOM^+^) TRN neurons that make up approximately four fifths of the cell population in the mouse TRN. In SOM^+^ TRN neurons, activation of ErbB4 normally reduces the strength of the glutamatergic drive that specifically arises from L6 CT neurons. However, in SOM-ErbB4 knockout (KO) mice, this reduction is lost resulting in an enhanced L6 CT→TRN drive and thereby an enhanced TRN→TC/TS inhibition. Complementing this genetic approach, mice were initially trained to make leftward or rightward movements to visual or auditory cues and were subsequently tested in a sensory discrimination task or in a rule-specific (“attend to vision”) discrimination task; correct responses were rewarded during training and testing. The sensory discrimination task contained “auditory/auditory” trials—a relevant (reward-associated) auditory stimulus among distracting auditory stimuli cued a leftward or rightward movement—and the rule-specific discrimination task contained “congruent” or “incongruent” trials—a relevant visual target and a previously relevant (now distracting) auditory stimulus cued the same (congruent) movement or opposite (incongruent) conflicting movements. Compared to wild-type (WT) mice, loss of ErbB4 in KO mice improves performance in auditory/auditory trials and impairs performance in visual/auditory incongruent trials—these changes in performance do not occur in KO mice when the enhanced L6 CT→TRN drive is reduced by blocking the postsynaptic delivery of the AMPA receptor subunit GluA4 in SOM^+^ TRN neurons. To account for the improved performance in auditory-auditory trials, enhanced TRN-mediated lateral inhibition of neurons responding to distracting stimuli in the ventral medial geniculate nucleus (vMGN)—containing first order TC neurons that convey auditory information from the inferior colliculus to auditory cortex—is proposed (Ahrens et al., 2015), whereas to account for the impaired performance in incongruent trials, TRN-mediated interactions between dLGN and vMGN (Ahrens et al., 2015)—producing enhanced cross-modal lateral inhibition—and between visTRN and audTRN (Ahrens et al., 2015; Makinson and Huguenard, 2015)—producing enhanced cross-modal disinhibition—are proposed. However, there is no evidence for such functional connections between dLGN and vMGN or between visTRN and audTRN.

Alternatively, the impaired performance of KO mice in visual/auditory incongruent trials (Ahrens et al., 2015) may specifically involve ILc neurons and striatal parvalbumin (PV) interneurons and an enhanced inhibition they receive from TRN neurons. Neurons in ILc are a major source of glutamatergic driver inputs to the striatum (Galvan and Smith, 2011; Smith et al., 2014), respond to the occurrence of behaviorally relevant (reward-associated) stimuli (Matsumoto et al., 2001; Minamimoto and Kimura, 2002; Minamimoto et al., 2005, 2014), and convey this information to cholinergic interneurons that are strategically distributed throughout the striatum (Graybiel et al., 1994; Smith et al., 2004; Galvan and Smith, 2011; Schulz and Reynolds, 2013). During classical conditioning in monkeys, the responses of these cholinergic interneurons are gradually modified by ILc driver inputs and reward-related nigrostriatal dopaminergic inputs (Aosaki et al., 1994a,b; Graybiel et al., 1994; Matsumoto et al., 2001)—this process constitutes an experience-dependent form of plasticity during sensorimotor learning. In turn, the cholinergic interneurons strongly modulate the activity of striatal output neurons (Pakhotin and Bracci, 2007; Ding et al., 2010; Schulz and Reynolds, 2013)—the spiny projection neurons (SPNs)—resulting in their development of response biases that facilitate the selection of future actions (Kimura et al., 2004; Graybiel, 2008; Minamimoto et al., 2009). It is important to note that the activity of striatal outputs can also be modulated by disynaptic TRN→PV interneuron→SPN pathways (Klug et al., 2018). Shaped and maintained by ILc and reward-related dopaminergic inputs, similar striatal response biases would be expected to develop in WT and ErbB4 KO mice trained to make reward-associated movements to visual and auditory cues. Thus, during the initial test session in visual/auditory incongruent trials (Ahrens et al., 2015)—when visual (relevant) and auditory (now irrelevant) stimuli cue conflicting movements—competing response biases of SPNs could largely account for the poor performance of WT mice (approximately 64% correct responses) and the even poorer performance of KO mice (approximately 40% correct responses) whose performance could be additionally hampered by an enhanced TRN-mediated lateral inhibition of ILc neurons responding to relevant cues as well as by an enhanced TRN-mediated inhibition of striatal PV interneurons accentuating previously acquired striatal response biases through disinhibition of SPNs. However, by the final test session, the performance of WT mice has markedly improved (approximately 86% correct responses) as has the performance of KO mice (approximately 70% correct responses). For WT and KO mice, their gradual improvements over test sessions (>1,000 trials) resemble inverted extinction curves and may reflect the gradual loss of an action-selection bias of SPNs to a previously rewarded but now unrewarded auditory cue.

### Internuclear Attentional Processes

Activation of neurons in one thalamic nucleus can lead to a temporary TRN-mediated interruption of the tonic firing of neurons in another thalamic nucleus (Crabtree and Isaac, 2002). Such intrathalamic activation-interruption sequences provide a mechanism for attentional control between competing sensory- and/or motor-related processes. Through TRN-mediated circuits, homologous regions in specific pairs of thalamic nuclei are interconnected and transmission through neurons in one of the two regions could be selected depending on the relative strength of the bottom-up salience or top-down relevance of the messages that are reaching the thalamus at any given moment. Thus, selection could occur between messages about ongoing sensory processes—through interactions among VPM, POm and ILc—ongoing motor processes—through interactions between VL and ILr—and ongoing sensory and motor processes—through interactions between ILc and ILr—and these selections would relate to different stages of information processing such as detection of events (ILc and ILr), detailed thalamic analyses (VPM and VL), or detailed cortical analysis (POm). Furthermore, through the wide range of known TRN-mediated intrathalamic interactions (Crabtree and Isaac, 2002), messages could be simultaneously selected representing multiple channels for attentional control in the thalamus. Concurrently, intrathalamic activation-interruption sequences could also provide a mechanism for selection between messages related to motor output processes. For example, descending axons from neurons in SC (Chevalier and Deniau, 1984) and from neurons in L5 cortex (Deschênes et al., 1994) convey motor instructions to motor regions of brainstem or spinal cord and branches of these axons carrying copies of these instructions (efference copies) are sent, respectively, to SC-recipient thalamic nuclei—for example, ILc—and thalamic nuclei containing higher order TC neurons—for example, POm. Because neurons in homologous regions of ILc and POm can interact, selection could alternate between messages about motor instructions implementing orienting responses—originating from SC—and motor instructions implementing goal-directed behaviors—originating from L5 cortex—thereby informing higher brain areas about ongoing shifts in motor output priorities through ILc and POm efferents.

In summary, TRN is involved in attentional processes that select sensory and motor information through bottom-up, top-down, and internuclear mechanisms. Of the two firing modes exhibited by thalamic neurons, tonic and burst firing (Jahnsen and Llinás, 1984; Kim and McCormick, 1998; Sherman, 2001), attentional mechanisms are often expressed by the modulation of tonic firing of TRN neurons—the possible modulation of their burst firing during these processes has yet to be determined. For messages that reach the thalamus, selection of information depends on their salience in bottom-up attentional control, their reward-associated relevance in top-down attentional control, and their salience or relevance in internuclear attentional control. During internuclear attentional processes, an increase in tonic firing of TRN neurons would occur—in response to glutamatergic driver inputs from neurons in one thalamic nucleus, an increase in activity and inhibitory output of TRN neurons enables them to temporarily interrupt the tonic firing of neurons in another thalamic nucleus. Conversely, during bottom-up and top-down attentional processes, TRN neurons play a key role in increasing the magnitude, or gain, of the sensory responses of TC neurons. Thus, the amount of GABA released by ssTRN neurons and the tonic firing of visTRN neurons are reduced, respectively, in bottom-up and top-down processes resulting in a reduction of these cells’ inhibition of TC neurons. During top-down attentional control, reduced firing of visTRN neurons occurs in a time-dependent manner during a stimulus anticipation period; prefrontal cortex is strongly influential in this process but does not directly affect some TRN sectors (e.g., visTRN). Furthermore, the strength of the feedforward L6 CT→TRN→TC/TS inhibition (Figure 2)—regulated by ErbB4-containing SOM^+^ TRN neurons—normally allows top-down attentional processes to effectively switch between sensory modalities depending on current behavioral goals. Because the various attentional processes can potentially engage an entire sensory or motor representation within TRN, large numbers of neurons could operate within the various TRN sectors to meet this requirement—for example, in top-down attentional processes, SOM^+^ neurons expressing ErbB4 constitute approximately 80% of the cell population in the mouse TRN.

## Conclusions and Future Directions

The studies surveyed in this review identify a diverse array of functional neuronal subnetworks in TRN. Their presence has been revealed using various combinations of anatomical, electrophysiological, genetic/molecular, and behavioral techniques. For some of these subnetworks, clarification of the distributions of their constituent neurons is needed, such as the distributions that mediate intrathalamic interactions among neurons in various groups of thalamic nuclei (Figure 3D; Crabtree and Isaac, 2002). TRN-mediated intrathalamic interactions among other groups of thalamic nuclei most likely exist and have yet to be revealed (e.g., between VA and MD suggested earlier). With one exception (McAlonan et al., 2008), the identified TRN subnetworks have been demonstrated in rodents; so, comparative studies of other mammalian species are also needed. Even so, in the monkey (McAlonan et al., 2008) and mouse (Halassa et al., 2014; Wimmer et al., 2015; Chen et al., 2016), top-down attentional control involves a TRN subnetwork that employs a similar functional mechanism, indicating that at least one such subnetwork is conserved across mammals (Schmitt and Halassa, 2017). Pending further interspecies comparisons, our current view of TRN subnetworks indicates considerable functional versatility among TRN neurons.

ErbB4, an NRG1 receptor expressed in SOM^+^ TRN neurons, regulates the strength of the L6 CT→TRN glutamatergic drive and hence the strength of TRN-mediated inhibition in thalamic nuclei (Ahrens et al., 2015). Differential NRG1 activation of ErbB4 receptors enables TRN neurons to efficiently switch between intramodality and intermodality attentional processes according to behavioral demands. This attentional flexibility suggests functional versatility among TRN neurons. Loss of ErbB4 receptors in TRN neurons markedly alters their flexibility and surprisingly, for such a basic cognitive function as attention, this loss does not appear to trigger cellular/molecular compensatory mechanisms; attentional flexibility is only restored after a further experimental manipulation that blocks trafficking of the AMPA receptor GluA4 subunit in SOM^+^ TRN neurons. However, there could be a circuit-based compensatory mechanism involving an interaction between enhanced strength of TRN-mediated inhibition (due to loss of ErbB4) and previous reward history—a steady improvement in performance over test trials in a task requiring intermodality attentional switching is consistent with the idea of a gradual extinction of an earlier action-selection bias mediated by BG circuits. Further work is required to test this and other possible compensatory mechanisms that allow substantial behavioral recovery following loss of NRG1/ErbB4 signaling in TRN neurons.

Functionally distinct neural subnetworks can occupy different TRN sectors as defined by different neuronal firing patterns during wakefulness and sleep (Halassa et al., 2014; Chen et al., 2016). Furthermore, connectivity with first order TC neurons in either sensory- or motor-related thalamic nuclei provides a possible anatomical basis for this functional heterogeneity between TRN sectors. In turn, these connections suggest a fundamental sensory-motor dichotomy among TRN neural subnetworks with different state-dependent activity profiles. This hypothesis needs to be comprehensively tested by examining the functional characteristics of more populations of TRN neurons and their various connections not only with other types of thalamic projection neurons—higher order and driver-like-recipient projection neurons—but also with a broad assortment of thalamic nuclei, particularly those involved in motor functions. Firmly establishing a sensory-motor dichotomy among TRN subnetworks would provide a valuable insight into the functional organization of the thalamus.

Functionally distinct neuronal subnetworks in TRN are often widely distributed in sensory and motor sectors. Two such subnetworks are made up of either elongated or broad AZ-related neural clusters that provide convergent inhibitory inputs onto neurons in various thalamic nuclei (Lam and Sherman, 2005, 2007, 2015). According to their efferent connections, elongated clusters are topographically organized, providing local modulation of first order TC neurons, and broad clusters are non-topographically organized, providing more global modulation of higher order TC neurons. The widespread distributions of TRN neurons in these two subnetworks can be thought of as forming templates, which possess the necessary anatomical and physiological properties that could guide the operations of other subnetworks such as those engaged in attentional processes. TRN subnetworks have been demonstrated to mediate bottom-up and top-down attentional mechanisms involving first order TC neurons, indicating that the TRN subnetwork of elongated neural clusters mediates these mechanisms (Figure 3C). Such mediation suggests considerable functional versatility among TRN neurons as their inhibitory outputs would rapidly adjust to meet attentional demands through axonal terminal mechanisms—in bottom-up attentional processes—or in response to cortical inputs—in top-down attentional processes. Whether AZ-related neural clusters in TRN similarly mediate attentional processes involving higher order TC neurons—through broad clusters—and driver-like-recipient TC/TS neurons—through elongated or broad clusters—remains a major challenge for future investigation.

## Author Contributions

JC wrote the manuscript and constructed the figures.

## Conflict of Interest Statement

The author declares that the research was conducted in the absence of any commercial or financial relationships that could be construed as a potential conflict of interest.
